# The effect of β-cell dysfunction on reproductive outcomes of PCOS undergoing IVF or ICSI embryo transfer cycles: a retrospective cohort study

**DOI:** 10.3389/fendo.2024.1327041

**Published:** 2024-03-05

**Authors:** Wenle Huang, Chang Liu, Lin Ding, Yan Li, Haisu Zhou, Shuwei Wang, Haiyan Yang

**Affiliations:** ^1^ Department of Obstetrics and Gynecology, Wenzhou Central Hospital, Wenzhou, Zhejiang, China; ^2^ Reproductive Medicine Center, The First Affiliated Hospital of Wenzhou Medical University, Wenzhou, Zhejiang, China; ^3^ Department of Endocrinology, The Second Affiliated Hospital of Shandong University of Traditional Chinese Medicine, Jinan, China; ^4^ Department of Endocrinology and Metabology, The First Affiliated Hospital of Shandong First Medical University & Shandong Provincial Qianfoshan Hospital, Jinan, Shandong, China; ^5^ Department of Anesthesia, Zhongshan Hospital, Fudan University, Shanghai, China

**Keywords:** β-Cell dysfunction, insulin resistance, hyperinsulinemia, polycystic ovary syndrome, IVF outcome

## Abstract

**Objective:**

To investigate the effects of β-cell dysfunction on IVF outcomes in women with PCOS.

**Methods:**

This retrospective cohort study includes 1,212 women with PCOS undergoing their first IVF cycle between September 2010 and December 2019. Beta-cell dysfunction was measured by homeostasis model assessment of β-cell function (HOMA-β) index.

**Results:**

In quartiles of HOMA-β, the incidence of miscarriage dramatically increased from 10.2% (Q1) to 31.1% (Q4) (*P*_for trend_ <0.001). Likewise, the incidence of miscarriage in quartiles of HOMA-β also showed a similar trend (*P*_for trend_ <0.001). After adjusting for confounding factors, logistic regression analyses showed that high HOMA-IR values were independently associated with a high risk of miscarriage, with the odds ratios (OR) and 95% confidence intervals for quartiles 2–4 versus quartile 1 were 1.30 (0.69-2.46), 1.82 (0.97-3.43), and 3.57 (1.86-6.85), respectively (*P*_for trend_ <0.001). When analyzed jointly, women in the highest HOMA-IR and highest HOMA-β group exhibited the highest risk for miscarriage compared with all other groups. Furthermore, higher HOMA-IR values were associated with higher risks of miscarriage among PCOS women regardless of HOMA-β values.

**Conclusions:**

β-cell dysfunction is independently associated with increased miscarriage rate and decreased live birth rate in women with PCOS. It also plays a synergistic role with IR in terms of the reproductive outcomes, while the influence of IR overweighs that of β-cell dysfunction.

## Introduction

Polycystic ovary syndrome (PCOS) is one of the most common endocrine disorders characterized by oligo-anovulation, hyperandrogenism and polycystic ovarian morphology, affecting 5%–18% women of reproductive age (1). Most women with PCOS may experience irregular menstruation, metabolic disorders, hirsutism and infertility (2). Therefore, subfertility has become a growing problem and there is an increased use of in vitro fertilization (IVF) as a last resort in women with PCOS (3). Moreover, compared to non-PCOS, PCOS is associated with increased risk of adverse pregnancy outcomes, including miscarriage (1.7-fold higher), gestational hypertension (2-fold higher), preeclampsia (4-fold higher), gestational diabetes (3-fold higher) and premature delivery (2-fold higher) (4–7). Hitherto, the pathogenesis of adverse pregnancy outcomes of PCOS has not yet been fully elucidated, making it more difficult to perform interventions and improve pregnancy outcomes.

Approximately 45%-65% of women with PCOS have insulin resistance (IR), which is considered an initiating factor and plays a key role in the development of PCOS (8, 9). It has been confirmed that IR is closely related to adverse pregnancy outcomes (especially increased risk of miscarriage) in women with PCOS undergoing IVF treatment (10, 11). In contrast to IR, relatively fewer studies have explored the effects of β-cell function (insulin secretion) on metabolic and pregnancy outcomes in women with PCOS. Our previous study indicated that both IR and β-cell dysfunction independently affected cardiometabolic abnormalities including obesity, central obesity, dyslipidemia and high blood glucose in women with PCOS. IR was also correlated with a higher prevalence of cardiometabolic abnormalities than β-cell dysfunction (76.7% vs. 61.2%) (12). These results indicate the different roles of IR and β-cell dysfunction in the development of cardiometabolic disorders in PCOS. However, it remains unknown whether these two pathological states exhibit different effects on pregnancy outcomes. Thus, in the present retrospective cohort study, we aimed to investigate the effects of β-cell dysfunction on IVF outcomes in women with PCOS.

## Patients and methods

### Participants

Initially, a total of 1,515 infertile women with PCOS undergoing their first in-vitro fertilization embryo transfer (IVF-ET) cycle from September 2010 to December 2019 at the Reproductive Center of the First Affiliated Hospital of Wenzhou Medical University were enrolled in this study. The diagnostic criteria included two out of three following features according to the 2003 Rotterdam diagnostic criteria (13): (1) menstrual abnormalities, including oligomenorrhea or amenorrhea; (2) clinical and/or biochemical hyperandrogenism, including hirsutism (Ferriman-Galwey score >6) or testosterone concentration >2.6 nmol/L; and (3) polycystic ovarian morphology under B-ultrasound as indicated by the number of follicles with a diameter of 2-9 mm ≥12 and/or ovarian volume ≥10 ml. The exclusion criteria were as follows: women older than 40 years of age (n=7); women with a history of thyroid dysfunction n=19), hyperprolactinemia (n=33), hydrosalpinx (n=109), endometriosis (n=39), adenomyosis (n=30), chromosome abnormality (n=20), pituitary microadenoma (n=1), recurrent spontaneous abortion (n=24) and uterine malformation (n=10); and women lost to follow-up during pregnancy (n=11). Finally, 1,212 women with PCOS were included in the study analyses. This study was approved by the Medical Ethics Committee of the First Affiliated Hospital of Wenzhou Medical University (Wenzhou, China) (2021R05), and approved a waiver of patient consent for the reason that all data were deidentified in this retrospective study.

### Study procedures

All patients received a standardized ovarian stimulation protocol (GnRH antagonist protocol or long GnRH agonist protocol), oocyte retrieval, fertilization, and embryo transfer. The GnRH antagonist protocol or long GnRH agonist protocol used in our reproductive center has been previously described (14). Good-quality embryos at cleavage stage were defined according to the Istanbul consensus with <10% fragmentation, stage-specific cell size and no multimucleation (15). Due to the elective single-embryo transfer policy, no more than two embryos have been transferred since June 2016. Luteal supportive therapy was administered orally with dydrogesterone (20 mg daily) and vaginally with progesterone (90 mg daily), starting on the day of oocyte retrieval, and was continued until 8 weeks of gestation.

### Definitions of β-cell dysfunction and insulin resistance

Beta-cell function was estimated by the homeostasis model assessment of β-cell function (HOMA-β) index as follows: HOMA-β=(20×FINS)/(FBG-3.5) (16). Beta-cell dysfunction was defined as HOMA-β in the top quartile (HOMA-β >186.86). IR was estimated by HOMA-IR index as follows: HOMA-IR=fasting blood glucose (FBG, mmol/L) x fasting insulin (FINS, mIU/L)/22.5 (17). The top quartile of HOMA-IR, which was greater than 3.75 in the present study, was defined as insulin resistance.

### Laboratory testing

Blood samples were acquired after overnight fasting for at least 8 hours. Levels of FBG, FINS and gonadal hormones were quantified by chemiluminescence. Concentrations of total cholesterol (TC), triglyceride (TG), low-density lipoprotein (LDL) and high-density lipoprotein (HDL) were measured by a dry slide enzymatic colorimetric assay. Serum LH, FSH, E2, testosterone and AMH were measured using an ultrasensitive enzyme-linked immunosorbent assay (ELISA) (Unicel Dxl 800, Beckman Coulter, USA). Fasting plasma glucose, total cholesterol (TC), serum triglycerides (TG), high-density lipoprotein (HDL) and low-density lipoprotein (LDL) were quantified by an autoanalyzer (AU 5800, Beckman, USA). The AMH data were limited due to the regular measurement of AMH in infertile women since June 2016 in our center. The intra-assay and inter-assay variations for the testing method were mentioned in our previous study (18). All measurements were performed at the First Affiliated Hospital of Wenzhou Medical University.

### Definitions of IVF outcomes

Biochemical pregnancy was defined as the detection of β-hCG in urine or serum after embryo transfer (19). Clinical pregnancy was defined as the presence of a gestational sac with fetal heart activity under ultrasound examination 35 days after embryo transfer (19). Spontaneous loss of an intra-uterine pregnancy prior to 22 completed weeks of gestational age was defined as miscarriage (19). A live birth was defined as baby born after 22 weeks of gestational age (19). The definition of the clinical pregnancy rate was the number of clinical pregnancies per 100 embryo transfer cycles (19). The miscarriage rate was defined as the number of spontaneous fetal loss per 100 clinical pregnancy cycles (19). The live birth rate was defined as the number of deliveries per 100 embryo transfer cycles (19). All IVF outcomes were obtained through electronic medical records.

### Statistical analysis

SPSS 23.0 software was used for all statistical analyses in the study. We divided the distribution of HOMA-β values into four groups from the lowest quartile (quartile 1, Q1) to the highest quartile (quartile 4, Q4). Participants were analyzed according to the quartile groups of HOMA-β. Demographic and biochemical variables with a skewed distribution were presented as the medians (interquartile ranges) according to quartiles of HOMA-β. *P* values for trends across all quartiles were calculated by linear regression analysis for continuous variables. Nonnormal distributed data were logarithmically transformed prior to linear regression analysis. Logistic regression analysis was performed to obtain the odds ratios for IVF outcomes based on quartiles of HOMA-β after adjusting for relevant variables (log-transformed). Meanwhile, *P* values for trends across quartiles were calculated by the Cochran–Mantel–Haenszel method. Odds ratios (ORs) and the corresponding 95% confidence intervals (CIs) were calculated in three models for the logistic regression analyses. Adjustments were made for the following variables: no variable was adjusted in model 1; age and BMI were adjusted in model 2; and SBP, HOMA-IR, TC, TG, basal T levels and the number of transferred embryos were further adjusted in model 3 based on model 2. Interaction analysis of HOMA-IR and HOMA-β on IVF outcomes was adjusted for age, BMI, SBP, TG, TC, basal T levels and the number of transferred embryos. ORs (95% CIs) of miscarriage and live birth were used to compare the combined effects of HOMA-IR and HOMA-β between different groups. All *P* values were two-sided, and *P*<0.05 was considered statistically significant.

## Results

### Baseline characteristics according to quartiles of HOMA-β in PCOS

Among the 1,212 infertile women with PCOS in the present study, the average age and the infertility duration was 29.69 and 3.81 years, respectively. There were 590 participants diagnosed with primary infertility with a prevalence rate of 48.7%, while the overall prevalence of secondary infertility was 51.3%.

The baseline characteristics of women with PCOS according to the quartiles of HOMA-β were described in **Table 1**. Subjects with a lower HOMA-β presented elevation in basal LH, FSH, FBG and HDL, but decreases in BMI, blood pressure, basal T, TC, TG, LDL, HOMA-IR, compared to those with higher HOMA-β (**Table 1**). The IVF outcomes, including fertilization mode, the number of retrieved oocytes, mature oocytes, fertilized oocytes, embryos obtained, good-quality embryos, transferred embryos and endometrial thickness, showed no significant differences among the quartiles of HOMA-β.

**Table 1:** Baseline characteristics according to quartiles of HOMA-β in PCOS.

Variables	Quartiles of HOMA-β				
	Quartile 1	Quartile 2	Quartile 3	Quartile 4	*P* for trend
	302	304	303	303	
Ovarian stimulation date^a^, n (%)					0.24
Tertile 1	110 (27.2)	92 (22.8)	116 (28.7)	86 (21.3)	
Tertile 2	97 (24.0)	103 (25.5)	97 (24.0)	107 (26.5)	
Tertile 3	95 (23.5)	109 (27.0)	90 (22.3)	110 (27.2)	
Age (years)	30.00 (27.00-32.25)	29.00 (27.00-31.75)	30.00 (27.00-32.00)	29.00 (27.00-32.00)	0.13
Infertility duration (year)	3.00 (2.00-4.00)	3.00 (2.00-4.00)	3.00 (2.00-5.00)	3.00 (2.00-5.00)	0.11
Infertility type, n(%)					0.86
Primary	145 (48.0)	158 (52.0)	137 (45.2)	150 (49.5)	
Secondary	157 (52.0)	146 (48.0)	166 (54.8)	153 (50.5)	
Fertilization mode, n(%)					0.92
IVF	200 (66.2)	197 (64.8)	199 (65.7)	202 (66.7)	
ICSI	80 (26.5)	79 (26.0)	76 (25.1)	75 (24.8)	
Half-ICSI	22 (7.3)	28 (9.2)	28 (9.2)	26 (8.6)	
BMI (kg/m2)	20.36 (18.80-22.87)	22.62 (20.89-24.65)	24.61 (22.08-27.34)	25.30 (23.05-27.34)	<0.001
SBP (mmHg)	110.00 (102.75-118.00)	110.00 (103.00-123.00)	118.00 (107.75-128.00)	120.00 (112.00-129.00)	<0.001
DBP (mmHg)	70.00 (66.00-79.00)	71.00 (68.00-79.00)	75.00 (70.00-81.00)	78.00 (70.00-83.00)	<0.001
Basal LH (IU/L)	6.78 (5.07-9.30)	7.57 (5.43-10.20)	7.18 (4.74-9.24)	6.20 (4.34-9.25)	0.10
Basal FSH (IU/L)	6.62 (5.65-8.10)	6.63 (5.73-7.60)	6.54 (5.51-7.56)	6.26 (5.29-7.30)	<0.001
LH/FSH ratio	0.99 (0.76-1.36)	1.10 (0.80-1.58)	1.04 (0.75-1.46)	1.09 (0.71-1.48)	0.65
Basal E2 (pmol/L)	148.50 (97.00-200.25)	161.00 (119.00-216.00)	159.00 (108.00-214.00)	150.00 (109.00-197.00)	0.83
Basal T (nmol/L)	1.64 (1.15-1.99)	1.86 (1.49-2.30)	1.79 (1.41-2.26)	2.09 (1.56-2.43)	<0.001
Basal AFC, n	25 (20-31)	27 (21-33)	25 (20-31)	27 (20-34)	0.24
AMH (ng/mL)*	8.04 (6.05-10.62)	8.56 (6.29-10.98)	8.90 (6.71-11.69)	7.80 (6.07-10.52)	0.99
FBG (mmol/L)	5.30 (5.00-5.60)	5.20 (5.00-5.50)	5.20 (5.00-5.60)	5.10 (4.80-5.40)	<0.001
FINS (mIU/L)	4.96 (3.69-6.41)	9.23 (7.79-10.85)	13.21 (11.27-16.04)	19.91 (15.55-25.44)	<0.001
TC (mmol/L)	4.49 (3.98-5.09)	4.72 (4.17-5.31)	4.76 (4.23-5.35)	4.89 (4.34-5.49)	<0.001
TG (mmol/L)	0.98 (0.72-1.34)	1.28 (0.85-1.63)	1.33 (0.95-1.91)	1.88 (1.28-2.45)	<0.001
HDL (mmol/L)	1.41 (1.23-1.65)	1.32 (1.15-1.52)	1.28 (1.15-1.45)	1.16 (1.00-1.36)	<0.001
LDL (mmol/L)	2.44 (2.06-2.93)	2.72 (2.24-3.26)	2.69 (2.20-3.20)	2.81 (2.39-3.35)	<0.001
HOMA-IR	1.14 (0.86-1.54)	2.15 (1.77-2.64)	3.08 (2.49-3.85)	4.46 (3.31-5.88)	<0.001
HOMA-β	55.81 (43.61-70.95)	105.43 (91.40-119.57)	156.34 (140.70-172.56)	248.18 (215.72-299.68)	<0.001
No. of retrieved oocytes	12.00 (8.00-16.00)	12.50 (8.00-17.00)	11.00 (7.00-15.00)	12.00 (8.00-16.00)	0.64
No. of mature oocytes	10.00 (6.00-13.00)	10.00 (7.00-14.00)	9.00 (6.00-13.00)	10.00 (6.00-13.00)	0.21
No. of fertilized oocytes	7.00 (4.00-10.00)	7.00 (5.00-11.00)	7.00 (4.00-10.00)	7.00 (4.00-10.00)	0.14
No. of embryos obtained	7.00 (3.00-9.00)	7.00 (4.00-10.00)	6.00 (4.00-10.00)	7.00 (3.00-9.00)	0.17
No. of good-quality embryos	2.00 (1.00-5.00)	3.00 (1.00-5.00)	3.00 (1.00-5.00)	2.00 (1.00-5.00)	0.69
No. of transferred embryos	2.00 (2.00-2.00)	2.00 (2.00-2.00)	2.00 (2.00-2.00)	2.00 (1.00-2.00)	0.10
Endometrial thickness (mm)	10.0 (8.45-11.00)	10.0 (8.50-12.00)	10.0 (9.00-11.00)	10.0 (8.00-12.00)	0.93

The quartile ranges of HOMA-β were < 82.28 (n=302), 82.28-129.54 (n=304), 129.55-188.75 (n=303), and > 188.75 (n=303).

*Quartile 1: n=64, Quartile 2: n=114, Quartile 3: n=82, Quartile 4: n=108.

a Tertiles of controlled ovarian stimulation starting date are as follows: tertile 1, September 1, 2010 to April 29, 2014; tertile 2, April 30, 2014 to November 7, 2017; tertile 3, November 8, 2017 to December 31, 2019.

HOMA-β, homeostasis model assessment of β cell function; PCOS, polycystic ovary syndrome; BMI, body mass index; LH, luteinizing hormone; FSH, follicle stimulating hormone; E2, estradiol; T, testosterone; AFC, antral follicle count; AMH, anti-mullerian hormone; FBG, fasting plasma glucose; FINS, fasting insulin; TC, total cholesterol; TG, triglycerides; HDL, high-density lipoprotein; LDL, low-density lipoprotein; HOMA-IR, homeostasis model assessment of insulin resistance; SBP, systolic blood pressure; DBP, diastolic blood pressure.

### IVF outcomes based on quartiles of HOMA-β and different beta-cell function plus insulin levels in PCOS

**Figure 1:**
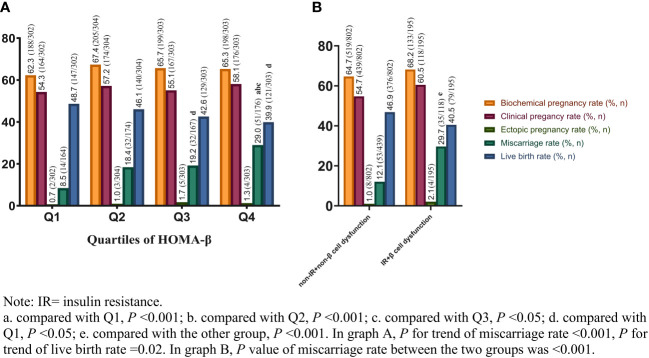
IVF outcomes based on quartiles of HOMA-β **(A)** and different beta-cell function plus insulin levels **(B)** in PCOS. IVF, in-vitro fertilization; HOMA-β, homeostasis model assessment of β cell function; PCOS, polycystic ovary syndrome.

**Figure 1A** showed the IVF outcomes based on quartiles of HOMA-β. In quartiles of HOMA-β, the miscarriage rate in Q4 was significantly higher than that in the other 3 quartiles (all *P* values <0.05). From the lowest quartile to the highest quartile of HOMA-β, the incidence of the miscarriage rate dramatically increased from 8.5% to 29.0% (*P*_for trend_ <0.001). However, the live birth rate decreased from Q1 to Q4 (*P*_for trend_ <0.05). No significant differences were observed in clinical pregnancy rate, biochemical pregnancy rate and ectopic pregnancy rate among the quartiles (*P >*0.05). The IVF outcomes in women with both IR and β-cell dysfunction (top quartile HOMA-IR and HOMA-beta) versus women without IR and without β-cells dysfunction were shown in **Figure 1B**. Compared with women without IR and β-cell dysfunction, the miscarriage rate in women with both IR and β-cell dysfunction was significantly higher (29.7% vs. 12.1%, *P <*0.001). Although the live birth rate in women with both IR and β-cell dysfunction was comparatively lower, no significant difference was found between the two groups (40.5% vs. 46.9%, *P >*0.05). Furthermore, the reproductive outcomes of women with and without resistance or with and without β-cell dysfunction were shown in **Table 2**. The miscarriage rate in women with IR or β-cell dysfunction was significantly higher than the controlled groups, while the live birth rate in women with IR was significantly lower than the non-IR group.

**Table 2:** Reproductive outcomes in women with and without β-cell dysfunction or with and without insulin resistance.

	β-cell dysfunction	Non-β-cell dysfunction	*P*-value
Biochemical pregnancy rate, n (%)	198/303 (65.3)	592/909 (65.1)	0.94
Clinical pregnancy rate, n (%)	176/303 (58.1)	505/909 (55.6)	0.44
Ectopic pregnancy rate, n (%)	4/303 (1.3)	10/909 (1.1)	0.76
Miscarriage rate, n (%)	51/176 (29.0)	78/505 (15.4)	<0.001
Live birth rate, n (%)	121/303 (39.9)	416/909 (45.8)	0.08
	IR	Non-IR	*P*-value
Biochemical pregnancy rate, n (%)	206/302 (68.2)	584/910 (64.2)	0.20
Clinical pregnancy rate, n (%)	184/302 (60.9)	497/910 (54.6)	0.06
Ectopic pregnancy rate, n (%)	6/302 (2.0)	8/910 (0.9)	0.12
Miscarriage rate, n (%)	60/184 (32.6)	69/497 (13.9)	<0.001
Live birth rate, n (%)	119/302 (39.4)	418/910 (45.9)	0.048

IR, insulin resistance.

### Odds ratios for IVF outcomes based on quartiles of HOMA-β in PCOS

**Table 3** lists the prevalence ratios for relevant IVF outcomes, including clinical pregnancy rate, biochemical pregnancy rate, ectopic pregnancy rate, miscarriage rate and live birth rate, according to quartiles of HOMA-β. With the first quartile of HOMA-β as the reference group, univariate logistic regression analysis showed significantly increased ORs for the prevalent miscarriage rate across HOMA-β categories (OR=4.37, 95% CI: 2.31-8.27) and the lowest odds ratio of live birth rate (OR=0.70, 95% CI: 0.51-0.97). After adjustment for traditional confounding factors (model 2), the ORs for the prevalent miscarriage rate, as compared with the lowest quartile, were 1.85 (95% CI, 0.90-3.80) for Q2, 2.13 (95% CI, 1.07-4.24) for Q3, and 3.09 (95% CI, 1.53-6.24) for Q4, respectively (*P*_for trend_ <0.001). Following further adjustment for SBP, TG, TC, basal T levels and the number of transferred embryos (model 3), a 94%, 78%, and 166% increase in ORs for the risk of the prevalent miscarriage rate was found in the second, third and fourth quartile, respectively, compared with those in the top one (*P*_for trend <_0.001). In terms of live birth rate in quartiles of HOMA-β, there was significant decreasing trend from Q1 to Q4 in model 1, 2 and 3 (*P*_for trend _ <0.05). Other IVF outcomes, such as the clinical pregnancy rate, biochemical pregnancy rate and ectopic pregnancy rate, were comparable in all 3 models in quartiles of HOMA-β.

**Table 3:** Odds ratios (OR) for IVF outcomes based on quartiles of HOMA-β in PCOS.

IVF outcomes	Quartiles of HOMA-β				
	Quartile 1	Quartile 2	Quartile 3	Quartile 4	*P* for trend
Biochemical pregnancy rate					
Model 1	1.00 (Reference)	1.26 (0.90-1.75)	1.16 (0.83-1.62)	1.14 (0.82-1.59)	0.54
Model 2	1.00 (Reference)	1.24 (0.88-1.75)	1.14 (0.80-1.63)	1.12 (0.77-1.62)	0.55
Model 3	1.00 (Reference)	1.31 (0.92-1.86)	1.19 (0.83-1.71)	1.16 (0.78-1.72)	0.39
Clinical pregnancy rate					
Model 1	1.00 (Reference)	1.13 (0.82-1.55)	1.03 (0.75-1.42)	1.17 (0.85-1.61)	0.47
Model 2	1.00 (Reference)	1.11 (0.80-1.55)	1.01 (0.71-1.42)	1.14 (0.80-1.62)	0.48
Model 3	1.00 (Reference)	1.20 (0.85-1.68)	1.06 (0.75-1.51)	1.23 (0.84-1.80)	0.35
Ectopic pregnancy rate					
Model 1	1.00 (Reference)	1.50 (0.25-9.01)	2.52 (0.49-13.07)	2.01 (0.37-11.04)	0.34
Model 2	1.00 (Reference)	1.46 (0.23-9.18)	2.53 (0.44-14.61)	1.96 (0.31-12.65)	0.34
Model 3	1.00 (Reference)	1.53 (0.24-9.78)	2.27 (0.40-13.04)	1.34 (0.21-8.63)	0.32
Miscarriage rate					
Model 1	1.00 (Reference)	2.41 (1.24-4.71)	2.54 (1.30-4.96)	4.37 (2.31-8.27)	<0.001
Model 2	1.00 (Reference)	1.85 (0.90-3.80)	2.13 (1.07-4.24)	3.09 (1.53-6.24)	<0.001
Model 3	1.00 (Reference)	1.94 (0.96-3.93)	1.78 (0.86-3.70)	2.66 (1.27-5.55)	<0.001
Live birth rate					
Model 1	1.00 (Reference)	0.90 (0.65-1.24)	0.78 (0.57-1.08)	0.70 (0.51-0.97)	0.02
Model 2	1.00 (Reference)	0.95 (0.68-1.32)	0.87 (0.61-1.23)	0.79 (0.55-1.13)	0.02
Model 3	1.00 (Reference)	1.04 (0.74-1.46)	0.92 (0.65-1.31)	0.91 (0.62-1.33)	0.04

Model 1 was unadjusted. Model 2 was adjusted for age and BMI. Model 3 was further adjusted for SBP, TG, HDL and basal T levels. All the confounding factors were log transformed. OR, odds ratio; IVF, in-vitro fertilization; HOMA-IR, homeostasis model assessment of insulin resistance; HOMA-β, homeostasis model assessment of β cell function; PCOS, polycystic ovary syndrome.

### Joint effects of HOMA-IR and HOMA-β on IVF outcomes

**Figure 2:**
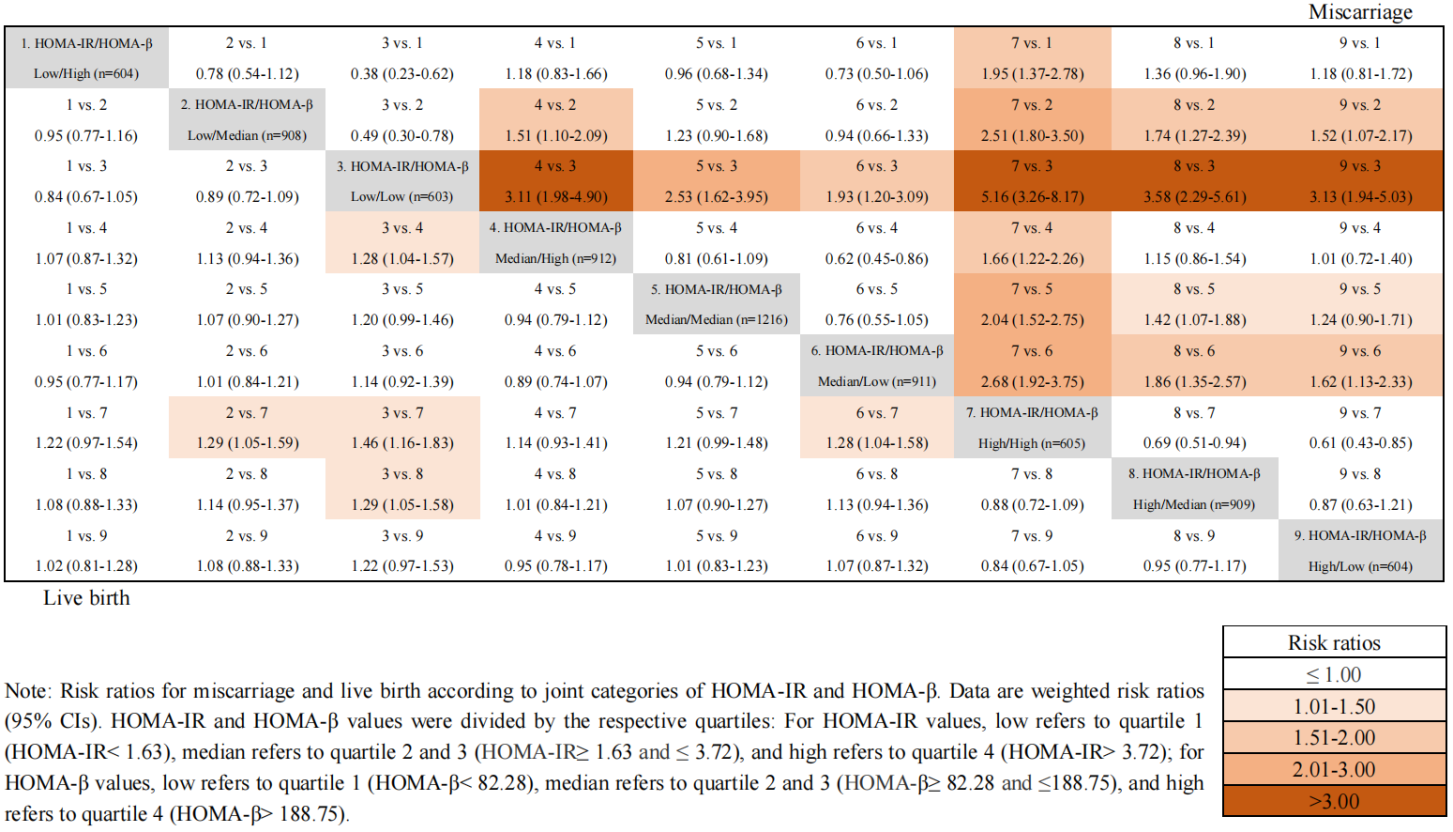
Joint effects of HOMA-IR and HOMA-β on IVF outcomes. Odds ratios for miscarriage and live birth according to joint categories of HOMA-IR and HOMA-β. Data are weighted odds ratios (95% CIs). HOMA-IR and HOMA-β values were divided by the respective quartiles: For HOMA-IR values, low refers to quartile 1 (HOMA-IR< 1.63), median refers to quartile 2 and 3 (HOMA-IR ≥1.63 and ≤ 3.72), and high refers to quartile 4 (HOMA-IR> 3.72); for HOMA-β values, low refers to quartile 1 (HOMA-β < 82.28), median refers to quartile 2 and 3 (HOMA-β≥ 82.28 and ≤188.75), and high refers to quartile 4 (HOMA-β> 188.75).

**Figure 2** showed the unadjusted odds ratios of miscarriage and live birth by comparing nine groups of patients with various combinations of HOMA-β and HOMA-IR values. Women with both high HOMA-β and high HOMA-IR values (group 7) exhibited the highest OR for miscarriage compared to all other groups. Furthermore, a higher HOMA-IR value (groups 7, 8 and 9) was associated with a relatively higher OR of miscarriage among participants regardless of the levels of HOMA-β values. However, the odds ratios of live birth in different groups according to the values of HOMA-IR and HOMA-β showed no significant differences after adjusting for confounding factors.

## Discussion

To the best of our knowledge, this is the first cohort study to explore the independent effect of β-cell dysfunction and the interaction effects of β-cell dysfunction and IR on the risks of IVF outcomes in women with PCOS. In the current study, we found that HOMA-β values were independently correlated with an increased risk of miscarriage.

Previous studies mainly have focused on the higher risk of miscarriage in PCOS women with insulin resistance (20, 21), while evidence regarding the effects of β-cell function on IVF outcomes is relatively sparse. Our results showed that high HOMA-β values were associated with an increased risk of miscarriage and a lower incidence of live birth, indicating that β-cell dysfunction (excess insulin secretion) independently exerted adverse reproductive effects on PCOS. The decrease in the HOMA-β values indicated a decrease in the sensitivity of human somatic cells to insulin receptors. Variation in β‐cell capacity is attributed to the growth of the β‐cell pool and insulin secretion ability (22). The balance between such a hyperdynamic β‐cell pool and insulin resistance ensures a steady flow of nutrients in women with PCOS when attempting conception (23). The exacerbation of IR leads to growing demands for insulin secretion by pancreatic β-cells. However, a hyperbolic relationship exists between IR and insulin secretion, that is with continued deterioration of IR, the compensation in insulin secretion could be limited with continued deterioration of IR (24). The resultant unfavorable state uncovers potential defects of β-cell function, thereby precipitating the development of gestational diabetes or type 2 diabetes in women with PCOS (25). Previous studies have demonstrated that the variation trend of HOMA-IR and HOMA-β is consistent in women with PCOS (12, 26), which agree with our findings. Therefore, it could be indicated that PCOS women with β-cell dysfunction are still at higher risks of miscarriage and lower incidence of live birth even without IR.

Furthermore, considering the classic feedback loop between hyperinsulinemia and IR, our study further examined the joint effects of β-cell dysfunction and IR on the miscarriage and live birth in women with PCOS. Our results suggested that women with both high HOMA-β and high HOMA-IR values exhibited the highest odds ratios for miscarriage and the lowest odds ratios for live birth. Additionally, according to various combinations of HOMA-β and HOMA-IR, the association of a lower HOMA-β value with the odds ratio of miscarriage was strengthened by a high HOMA-IR value, indicating that although both insulin resistance and β-cell dysfunction were closely associated with miscarriage, the effect of IR overweighed that of β-cell dysfunction. A recent study indicated that women with PCOS and IR might result in a higher risk of miscarriage, but did not impair live birth rate (27). It has been reported that the variation of HOMA-β is hyperbolic in the progression of diabetes and is highly interfered by insulin resistance, which increases the difficulty of determining whether the value of HOMA-β represents the compensation stage or decompensation stage (28). In this study, we found that women with IR has significantly lower live birth rate, which is not in accordance with previous findings. Moreover, although the live birth rate were comparable between the β-cell dysfunction and non-β-cell dysfunction, women with both high HOMA-β and high HOMA-IR values exhibited the lowest odds ratios for live birth after interactive analysis. This result indicates that the negative association between IR and adverse reproductive outcomes seemed to be amplified with more vulnerable β-cell function in women with PCOS.

When compared with women with both low values of HOMA-IR (HOMA-IR< 1.63) and HOMA-β (HOMA-β<82.28), women with both insulin resistance (HOMA-IR>3.72) and β-cell dysfunction (HOMA-β>188.75) have approximately 5.16-fold higher miscarriage rate, and 1.46-fold lower live birth rate. Therefore, from a clinical point of view, it could be hypothesized that after appropriate pretreatment to lower HOMA-IR and HOMA-β, the reproductive outcomes in women with PCOS might have improved significantly. These insights shine a light on the application of a novel therapy for PCOS with insulin-sensitizing drugs. Metformin, an insulin-sensitizing drug, has been widely applied in the pretreatment of women with PCOS undergoing IVF treatment during the past decades (29). Metformin administration could reduce the risk of ovarian hyperstimulation syndrome and miscarriage in women with PCOS, which provides a new idea to improve the reproductive outcomes in PCOS women with insulin resistance and β-cell dysfunction (30).

Several potential mechanisms account for the negative effects of β-cell dysfunction as well as IR on reproductive outcomes in women with PCOS. First, the high insulin level in peripheral blood leads to the abnormal secretion of insulin-like growth factor (IGF-1) and IGF-2. IGF facilitates the implantation of the human embryo in the endometrium during IVF and plays an important role in trophoblast morphogenesis and placental microvasculature (31, 32). Second, studies on PCOS-like rats indicate that IR causes the activation of ferroptosis in the gravid uterus and placenta. Furthermore, necroptosis and apoptosis might play a role in compensating or coordinating for IR-induced ferroptosis when the gravid uterine and placental dysfunction occur (33). In addition, hyperinsulinemia and IR can lead to increased secretion of reactive oxygen species, which further cause mitochondrial and placental dysfunction after pregnancy, thus increasing the risk of miscarriage and decreasing the live birth rate (34, 35).

To our knowledge, this study was unique in that we included women with PCOS undergoing their first IVF cycle and studied both the independent and combined effects of β-cell dysfunction and insulin resistance on IVF outcomes. The novelty of our study was the independent and joint effect of HOMA-β and HOMA-IR on reproductive effects. Considering that PCOS women with various combinations of β-cell dysfunction and IR might present different odds ratios for miscarriage, early screening and individualized intervention should thus be tailored. Therefore, even in PCOS women with mild hyperglycemia, glucose‐lowering interventions before IVF could improve pregnancy outcomes. In addition, the calculation of HOMA-IR and HOMA-β is easy and has been widely acknowledged, which anticipates a highly practical value of our findings in the clinical practice. However, the present study had several limitations and should be taken into consideration. First, this is a retrospective study that looks back for a long span of time in which clinical practice, biochemical measurements, IVF treatments might have changed dramatically. In order to minimize the potential bias, we analyzed the subjects from Q1 to Q4 according to the tertiles of ovarian stimulation date in **Table 1**. We found that there were no significant differences in subjects from Q1 to Q4 in terms of the grouping of ovarian stimulation date in both Tables. However, prospectively designed multi-centers studies, follow-up of newborns and the effect of medical pretreatment on reproductive outcomes are still needed for further evaluation. Second, glucose clamp has been undeniably recognized as the gold standard for evaluating insulin metabolism. However, they may be perceived as invasive and expensive for use in clinical studies with large samples. Thus, surrogate markers, such as HOMA, have been proposed as alternative markers for insulin sensitivity and secretion, which can be repeatable and reproducible in the same way as gold standards. Although validation studies have indicated tight correlations between the HOMA models and gold-standard methods, the findings should be interpreted carefully. Third, although carefully adjusted for a set of confounders in the analysis, unmeasured confounders, such as dietary factors and physical activity, may affect the results to some extent. In addition, since the subjects in the present study were Chinese women, these conclusions might not be directly applied to populations of other ethnicities.

## Conclusions

In summary, our findings indicate that β-cell dysfunction is independently associated with increased miscarriage rate and decreased live birth rate in women with PCOS. Furthermore, it also plays a synergistic role with IR in terms of the reproductive outcomes, while the influence of IR overweighs that of β-cell dysfunction. Therefore, early screening and interventions for β-cell dysfunction and IR in women with PCOS may be extremely helpful for improving conception opportunities.

## Data availability statement

The raw data supporting the conclusions of this article will be made available by the authors, without undue reservation.

## Ethics statement

The studies involving humans were approved by The First Affiliated Hospital of Wenzhou Medical University. The studies were conducted in accordance with the local legislation and institutional requirements. The ethics committee/institutional review board waived the requirement of written informed consent for participation from the participants or the participants’ legal guardians/next of kin because all the data were deidentified in this retrospective study.

## Author contributions

WH: Conceptualization, Formal Analysis, Methodology, Writing – original draft. CL: Conceptualization, Data curation, Formal Analysis, Investigation, Methodology, Writing – original draft, Writing – review & editing. LD: Data curation, Writing – review & editing. YL: Data curation, Writing – original draft. HZ: Formal Analysis, Software, Writing – original draft. SW: Formal Analysis, Writing – review & editing. HY: Conceptualization, Funding acquisition, Resources, Supervision, Validation, Writing – review & editing.
